# A method for phenotypic evaluation of grapevine resistance in relation to phenological development

**DOI:** 10.1038/s41598-023-50666-4

**Published:** 2024-01-09

**Authors:** Stefan Schumacher, Caroline Mertes, Thomas Kaltenbach, Gottfried Bleyer, René Fuchs

**Affiliations:** Department of Biology, State Institute of Viticulture and Oenology, Merzhauser Str. 119, 79100 Freiburg im Breisgau, Germany

**Keywords:** Plant breeding, Plant immunity

## Abstract

Fungus-resistant grapevine cultivars, so called PIWIs, are characterized by increased resistance to powdery mildew and downy mildew. However, in order to maintain the durability of resistance in these new grape cultivars, targeted fungicide treatments are recommended. For ideal schedule of these treatments, it is necessary to recognize the most sensitive organs of the grape. This study introduces a method for phenotypic evaluation of *Plasmopara viticola* resistance in grape clusters under controlled and standardized conditions during phenological development over the entire season. The approach was validated with the traditional cultivar Pinot Noir and the PIWIs Cabernet Cortis (*Rpv3.3*, *Rpv10*), Solaris (*Rpv3.3*, *Rpv10*) and Souvignier Gris (*Rpv3.2*). All cultivars were susceptible during the early stages of development up to flowering, and resistance levels increased as phenological development progressed. Cabernet Cortis and Solaris clusters were susceptible until fruit development (BBCH 71–73) when they became almost completely resistant. No differences between Souvignier Gris and Pinot Noir were detected until berries were pea-sized (BBCH 75) when *P. viticola* resistance of Souvignier Gris clusters increased significantly. Ontogenetic resistance in Pinot Noir was detected at berry touch (BBCH 77–79) and clusters of this cultivar were almost completely resistant at the beginning of ripening (BBCH 81–83). These results indicate that the approach presented is suitable for determining the resistance of grape cultivars at different stages of development. Consequently, in the future, fungicide applications can be adjusted more precisely to the resistance level of a grape cultivar during the growing season.

## Introduction

The genus *Vitis* consists of 83 accepted species^1^. The best-known representative of this genus is the species *Vitis vinifera*, subdivided into the wild grape *V. vinifera* ssp. *sylvestris* and the cultivated *V. vinifera* ssp. *vinifera*^2^. *V. vinifera* ssp. *vinifera* has been used for thousands of years for both table grape and wine production and is now cultivated around the entire world^3^. While *V. vinifera* is native to Europe *Plasmopara viticola,* the pathogen responsible for the major viticultural disease grapevine downy mildew originates from the eastern North American continent and was introduced to Europe in the 1870s^4^. Consequently, *V. vinifera* lacks evolutionary defense mechanisms and is highly susceptible to downy mildew which may cause reduced fruit quality and complete yield loss in unprotected vineyards^5^. Especially in wine regions with humid climates, significant applications of fungicides are necessary to maintain plant health^6^.

Effective crop protection with minimized risk to humans and the environment is achieved by integrated pest management (IPM) and widely established in European agriculture^7^. Various approaches have been implemented in viticulture to minimize the use of fungicides^8^. Recovery and reutilization of pesticide that has not reached the foliage by modern application technology allows significant reduction of total quantities^9^. Furthermore, model based decision support systems like VitiMeteo offer disease risk forecasts and strategies for optimal pesticide application timing^10^. A further reduction of pesticide applications can be achieved by breeding and selecting planting material with increased innate resistance. In contrast to *V. vinifera,* American or East Asian wild species, such as *Vitis rupestris* or *Vitis amurensis* have developed powerful defense mechanisms during their coevolution with *P. viticola*^11^. To date, 33 QTL have been identified in wild *Vitis* spp. that confer resistance to downy mildew of grapevine, known as *Rpv* (*Resistance to P. viticola*) loci^12^. *Rpv1, Rpv3* (*Rpv3.1, Rpv3.2, Rpv3.3*), *Rpv10* and *Rpv12* are currently of relevance in viticultural breeding programs^13^. Classical breeding using American or Asian wild species and traditional European cultivars has resulted in numerous fungus-resistant grapevine cultivars, so-called PIWIs (German: pilzwiderstandsfähige Rebsorten). PIWIs combine the resistance mediating properties of wild species with the desirable oenological characteristics of *V. vinifera*^14^.

*Rpv*-mediated resistance can be overcome by adapted *P. viticola* isolates. For example, *Rpv3*-mediated resistance was overcome more than a decade ago in vineyards in France and Germany^15^. To reduce adaptation of pathogens, modern breeding programs combine multiple resistance loci in new cultivars, known as pyramiding^16^. In spite of multiple resistance loci, weather and infection conditions may call for fungicide treatments during the season to avoid pathogen adaptation to resistance^13,17^. Practical spraying recommendation should thus consider the fungicide reduction potential of different cultivars while maintaining yields. This requires advanced knowledge about seasonal resistance variations of different grapevine organs.

Studies on resistance mechanisms or resistance levels are usually carried out under standardized conditions in laboratory tests such as leaf disc assays^18–20^. To our knowledge, this is the first study considering different phenological stages of both leaves and clusters. Selection for disease resistance in grapevine breeding usually starts with marker-assisted selection (MAS) on the first seedlings and is monitored in the field over decades. This exposes the vines to natural infection pressure, which is highly dependent on the location and weather conditions. The alternative approach presented here enables breeders to investigate *P. viticola* resistance of cultivars during seasonal development from inflorescences to ripe fruit under standardized and controlled conditions. The procedure furthermore provides a tool for phenotyping resistance-loci as well as for the underlying mechanisms. The knowledge gained through extensive resistance phenotyping offers optimized decision support for plant protection strategies adapted to PIWIs.

## Material and methods

### Grapevine cultivars

The resistance of grape leaves and clusters was evaluated on *V. vinifera* cv. Cabernet Cortis, cv. Pinot Noir, cv. Solaris and cv. Souvignier Gris. The traditional grapevine cultivar Pinot Noir is highly susceptible to *P. viticola*. The PIWIs used in this study possess QTLs mediating partial resistance to grapevine downy mildew. The cultivar Souvignier Gris carries the single locus *Rpv3.2*, whereas Cabernet Cortis and Solaris harbor the combination of *Rpv3.3* and *Rpv10*^21,22^. All vines used were grafted on SO4.

42 potted plants per grape cultivar (pot size: 7 L; commercially available universal soil) were grown in an open greenhouse at natural ambient temperature in Freiburg im Breisgau, Germany. Drip irrigation was adapted to the weather. On average, during the growth season (from April to September), vines were watered for 10 min after exposure to 300 kilolux-hours but no later than 36 h after the last watering. The vines were fertilized once a week with 1.2 g NPK-fertilizer (Hakaphos^®^ Blau 15–10–15(+ 2), COMPO EXPERT GmbH, Germany) in 300 ml water. During the growing season, vines were treated once a week according to a standard regime with commercially available fungicides to avoid infections with grapevine powdery mildew. All vines were trained to grow with two fruit canes and were pruned after reaching a height of 2 m. After the growing season and completed leaf fall, fruit canes were cut back to two buds. For wintering, the vines remained in the open greenhouse. After first budding in spring, vines were protected from late frost to avoid frost damage. All grapevine plants were potted in 2018. These vines carried the first fruit in 2019 and were used for the experiments in the years 2020, 2021, 2022 and 2023.

All *Vitis vinifera* cultivars used are commercially available and experimental research, as well as field studies, were carried out in accordance with relevant institutional, national, and international guidelines and legislation complying with the IUCN Policy Statement on Research Involving Species at Risk of Extinction and the Convention on the Trade in Endangered Species of Wild Fauna and Flora.

### Pathogen material

*P. viticola* was collected from leaves of *V. vinifera* cv. Mueller-Thurgau plants from a vineyard in Freiburg im Breisgau, Germany. Consequently, a different field isolate of *P. viticola* was used every year. The pathogen was artificially propagated in the laboratory on potted *V. vinifera* cv. Mueller-Thurgau plants throughout the season to produce fresh sporangia for the inoculation of leaves and clusters. Sporulating leaves were rinsed with ddH_2_O to obtain a sporangia solution. The number of sporangia was determined with a Fuchs-Rosenthal counting chamber and diluted to a concentration of 40,000 sporangia/ml. After spray inoculation with a commercially available pump sprayer, potted plants were covered with a polyethylene bag to maintain high relative humidity overnight. Seven days after inoculation, infected vines were sprayed with water and once again covered with a polyethylene bag overnight to induce sporulation.

### Resistance assessment on grape clusters

Phenotypic differences in resistance between grape cultivars were assessed over seven stages during their phenological development by rating downy mildew disease severity. The following phenological stages according to the BBCH-scale were tested: BBCH 53–55 (inflorescences visible and start to enlarge), BBCH 57–59 (inflorescences are fully developed and flowers start separating), BBCH 65–68 (full flowering, 50–80% of the flower hoods are fallen), BBCH 71–73 (beginning of fruit development, berries are groat-sized), BBCH 75 (berries are pea-sized and bunches hang), BBCH 77–79 (berry touch) and BBCH 81–83 (beginning of ripening, berries develop color)^23^. Inoculation was performed in a modified climate chamber. Temperatures inside the chamber were kept above 17.5 °C and vines were spray irrigated automatically for 5 s every 2 h, resulting in a high relative humidity of nearly 100% during the whole infection period.

**Figure 1:**
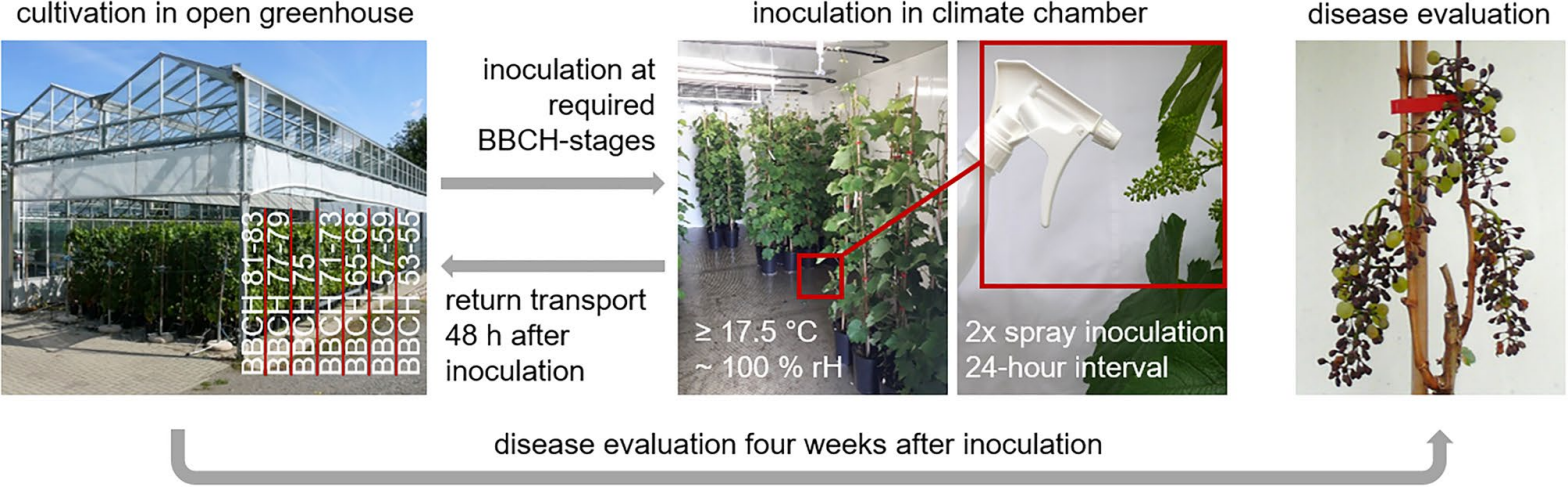
Schematic illustration of the trial procedure. After reaching the required phenological stage, inflorescences/clusters of six potted vines were sprayed twice with a *P. viticola* sporangia solution. Disease severity of downy mildew was rated individually for every cluster 4 weeks after inoculation.

After reaching the desired phenological stage, six potted vines of every cultivar were transferred from the open greenhouse to the chamber for inoculation (Fig. 1). Clusters were spray inoculated with a sporangia solution of 40,000 sporangia/ml with a commercially available pump sprayer until they were completely covered (dripping wet). Inoculations were performed twice with an interval of 24 h to induce a high infection pressure. 48 h after the first inoculation, vines were transferred back to the open greenhouse to ensure a natural phenological development thus assuring that every single plant was infected only at a single phenological stage throughout the season, and that all phenological stages were covered in each cultivar.

Evaluation of resistance levels was carried out by rating disease severity four weeks after inoculation for every phenological stage separately. Disease severity was visually rated for each cluster by determination of the percentage of infected berries on a 0–100% scale in accordance with the standards of the European and Mediterranean Plant Protection Organization (EPPO). The following gradations were selected for assessment: 0%, 1%, 5%, 10%, 20%, 30%, 40%, 50%, 60%, 70%, 80%, 90% or 100%. A total of 1856 inoculated grape clusters were thus evaluated in four independent trials.

### Leaf disc assay

Resistance of maturing leaves was assessed by disease severity ratings of leaf disk assays throughout the season. During inoculation of grape clusters three leaves from three different vines from each cultivar were detached from the plants in the climate chamber. By sampling leaves positioned next to the clusters, it was assured that leaves were of the same age as the clusters used for inoculation experiments.

Detached leaves were disinfected for 30 s with 70% ethanol, rinsed twice with distilled water and carefully dried with household tissue. Twelve leaf discs per leaf were then punched out using a cork borer (diameter 14 mm) and placed onto their adaxial surface on a 1% (w/v) water agar plate. Inoculation of leaf discs was carried out by pipetting 100 µl drops of a fresh prepared *P. viticola* sporangia solution (40,000 sporangia/ml) onto the abaxial leaf sides. The inoculation drop was removed after incubation for one night in the dark at 24 °C. The plates were hermetically closed (Parafilm M, Germany) and placed upside down in a climate chamber at 24 °C/18 °C (day/night; (16 h/8 h). In addition to the leaves sampled from the potted vines in the climate chamber, the third to fifth leaf from the top of a shoot of a greenhouse cv. Pinot Noir plant were used to assure the functionality of the sporangia solution used in a control leaf disc assay. Evaluation of disease severity was carried out seven days post inoculation based on a scale indicating the percentage of the area beneath the inoculation drop showing sporulation (1: 0%; 2: 1–7%, 3: 8–15%, 4: 16–30%, 5: 31–45%, 6: 46–60%; 7: 61–75%; 8: 76–90%, 9: 91–100%). Mean values were calculated from 36 leaf discs per cultivar and phenological stage, representing disease severity.

### Statistical analysis

Values for disease severity of downy mildew of grapevine obtained from ratings of inoculated grape clusters and leaves were statistically analyzed with GraphPad Prism 9 (Version 9.4.1). High levels of disease severity were used as an indicator for low levels of resistance. For determination of statistically significant differences in resistance between different phenological stages of clusters and leaves, values obtained from ratings of the specific stages were compared. Furthermore, resistance in clusters and leaves at a specific phenological stage was analyzed for statistically significant differences between the different cultivars. Data was tested for a normal (Gaussian) distribution using the Shapiro–Wilk normality test with a significance level of α = 0.05. Since none of the data was normally distributed, analysis for significance was performed by non-parametric Kruskal–Wallis one-way ANOVA. Multiple comparisons were done using Dunn’s test. Significant differences are indicated by different letters in the graphs if the calculated *P*-value between two groups was 0.05 or smaller.

## Results

### Resistance of grape clusters to *Plasmopara viticola* differs between phenological stages

**Figure 2:**
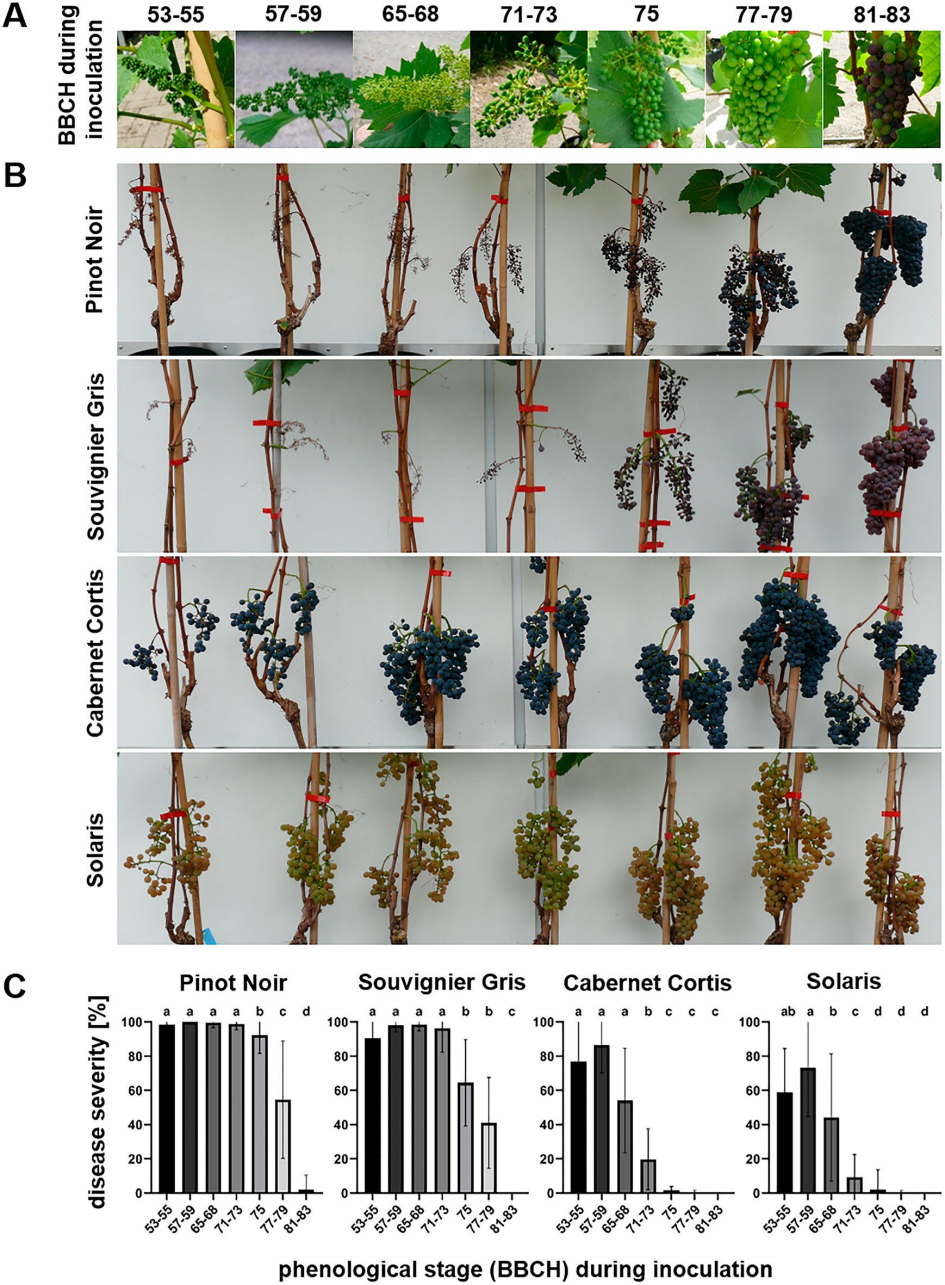
Downy mildew resistance of grape clusters increases in a cultivar dependent fashion during the phenological development. (**A**) Photographic pictures of inflorescences/clusters taken at different phenological stages used for inoculation. (**B**) Downy mildew symptoms on clusters inoculated at different phenological stages indicated in Fig. 2A. Photos show one out of 4 years (2020) with a similar result. Pictures were taken when fruit were ready for harvest (BBCH 89). (**C**) Disease severity rated on grape inflorescences/clusters from Pinot Noir (N = 518), Souvignier Gris (N = 367), Cabernet Cortis (N = 362) and Solaris (N = 609) 4 weeks after inoculation with *P. viticola* during the stages indicated in Fig. 2A. High levels of disease severity indicate low levels of resistance. Different colored bars show the average disease severity from four experiments performed during the years 2020, 2021, 2022 and 2023. Error bars show standard deviation, different letters indicate significant differences (Kruskal–Wallis-test followed by multi comparison with Dunn’s test, α = 0.05).

Resistance to *P. viticola* during fruit development was assessed with PIWI cultivars Cabernet Cortis, Solaris and Souvignier Gris as well as with the traditional cultivar Pinot Noir. To represent seasonal maturation of clusters, seven phenological stages were artificially infected, and disease severity was rated 4 weeks after inoculation (Fig. 2A–C).

The susceptible cultivar Pinot Noir showed a disease severity of almost 100% during the phenological stages up to and including BBCH 75. During the later BBCH 77–79 and BBCH 81–83 stages, disease severity decreased to 55% and 2%, respectively. Souvignier Gris was also highly susceptible to *P. viticola* with a disease severity of nearly 100% until BBCH 75 after which severity decreased to 64%. A further decline to 41% was visible at BBCH 77–79 while no typical downy mildew symptoms were observed with the onset of maturity (BBCH 81–83). Cabernet Cortis showed its lowest resistance during the first two phenological stages from BBCH 53–55 to BBCH 57–59 with a disease severity of 77% and 86%, respectively. Thereafter, the cultivars’ resistance increased as indicated by decreasing disease severity from 54% (BBCH 65–68) to 20% (BBCH 71–73) to 2% (BBCH 75). At berry touch (BBCH 77–79) and beginning of fruit ripening (BBCH 81–83), downy mildew symptoms were no longer visible. Solaris showed a pattern very similar to Cabernet Cortis. From the formation of inflorescences (BBCH 53–55) until flowering (BBCH 65–68) disease severity ranged between 44 and 73%. The strong increase in resistance at BBCH 71–73 was reflected in a disease severity of 9%. During the last three phenological stages until ripening, no visible symptoms were recorded (Fig. 2).

### Resistance of clusters to *P. viticola* at different phenological stages varies between cultivars

**Figure 3:**
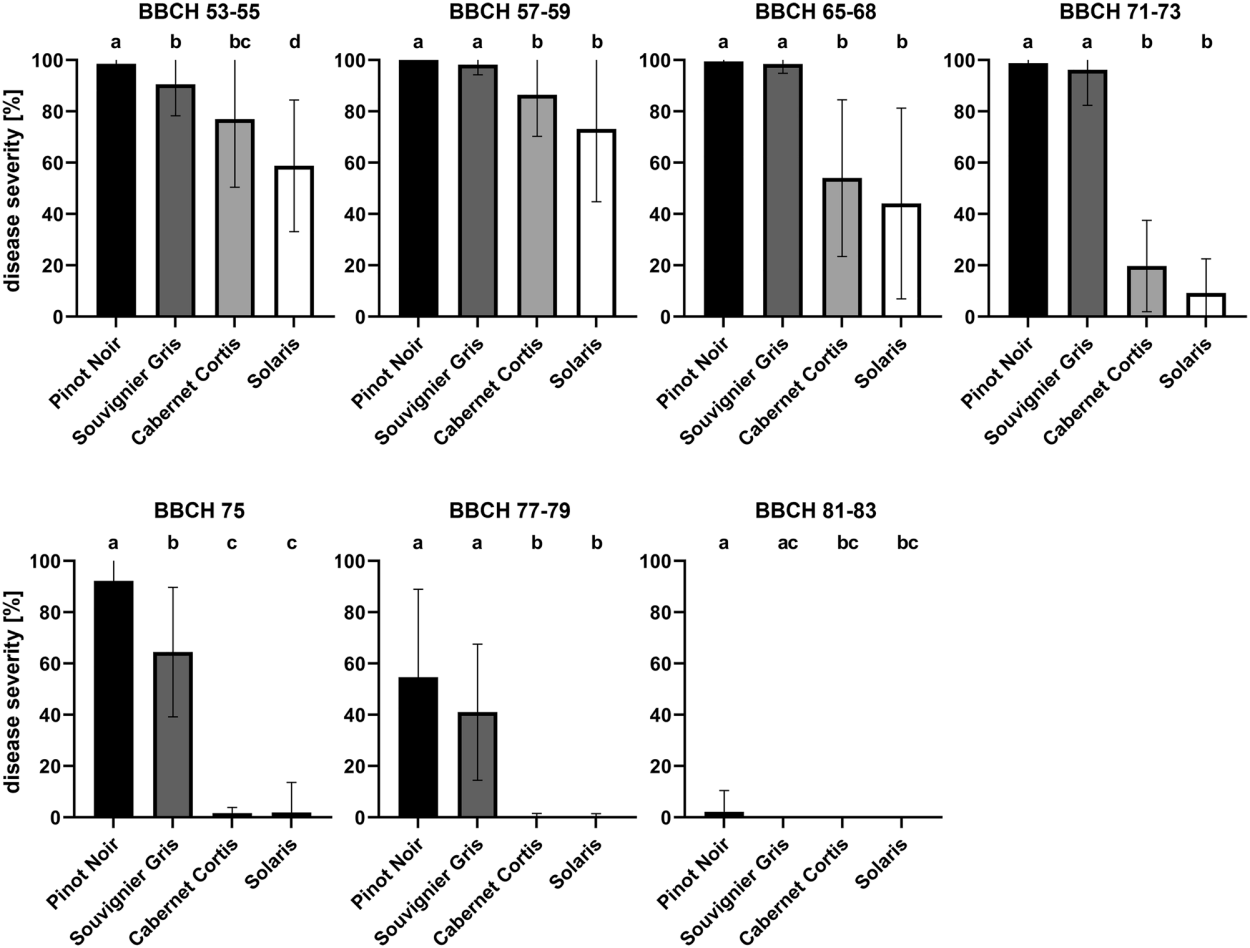
Resistance of grape clusters to *P. viticola* at different phenological stages differs between cultivars. Graphs show a comparison of disease severity on inflorescences/clusters of four grape cultivars at different stages of development (BBCH 53–55, N = 320; BBCH 57–59, N = 248; BBCH 65–68, N = 264; BBCH 71–73, N = 264; BBCH 75, N = 258; BBCH 77–79, N = 283; BBCH 81–83, N = 219). High levels of disease severity indicate low levels of resistance. Bars show the mean disease severity from 4 years (2020, 2021, 2022 and 2023). Error bars show standard deviation, different letters indicate significant differences (Kruskal–Wallis-test followed by multi comparison with Dunn’s test, α = 0.05).

Although resistance to downy mildew increased in all observed grape cultivars during their phenological development, differences in resistance between the cultivars were observed during the season (Fig. 3). The comparison revealed that Souvignier Gris, Cabernet Cortis and Solaris were less susceptible to *P. viticola* than Pinot Noir in the very first stage of inflorescence development (BBCH 53–55). At the next stage just before flowering (BBCH 57–59), Cabernet Cortis and Solaris proved less susceptible than Souvignier Gris and Pinot Noir, whose disease severity was similar (86% for Cabernet Cortis and 73% for Solaris). At flowering (BBCH 65–68), Cabernet Cortis and Solaris showed a disease severity of 54% and 44%, respectively as compared to nearly 100% for Pinot Noir and Souvignier Gris. The differences between individual cultivars became even more apparent with fruit development. With groat-sized berries at the time of inoculation (BBCH 71–73), disease severity reached only 20% for Cabernet Cortis and was even lower for Solaris (9%). In Pinot Noir and Souvignier Gris disease severity was still 100%. It was not until the BBCH 75 stage that the first differences between the cultivars Pinot Noir and Souvignier Gris became apparent. While disease severity was still very high in Pinot Noir (92%), it dropped to 65% in Souvignier Gris. The cultivars Cabernet Cortis and Solaris were already almost completely resistant at this point. As development progressed (BBCH 77–79), susceptibility of Pinot Noir and Souvignier Gris continued to decrease, as indicated by a disease severity of 55% and 41%, respectively. In the last phenological phase investigated corresponding to the beginning of ripening (BBCH 81–83), there were almost no more differences between the cultivars, i.e. all grape cultivars inoculated were completely resistant to *P. viticola*.

### Resistance level in leaves is higher than in clusters

**Figure 4:**
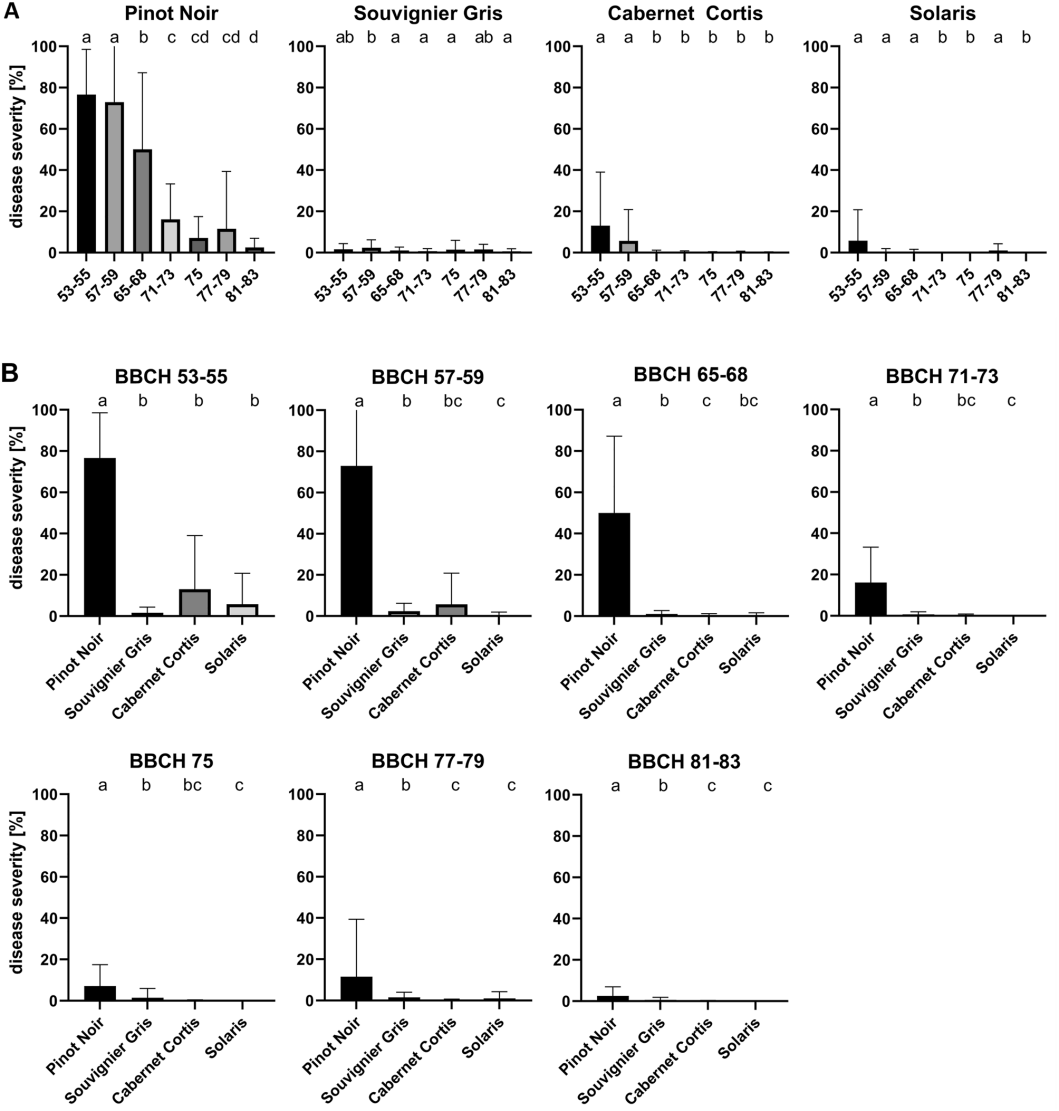
Leaves of PIWI cultivars show significantly higher resistance to *Plasmopara viticola* than leaves of Pinot Noir*.* (**A**) Graphs show mean disease severity obtained from leaf disc assays from Pinot Noir (N = 108), Souvignier Gris (N = 108), Cabernet Cortis (N = 108) and Solaris (N = 108) by scoring sporulation seven days after inoculation with *P. viticola*. High levels of disease severity indicate low levels of resistance in the leaf discs. (**B**) Comparison of disease severity on leaves at different stages of development. Different colored bars show the average disease severity from three experiments performed during the years 2021, 2022 and 2023. Error bars show standard deviation, different letters indicate significant differences (Kruskal–Wallis-test followed by multi comparison with Dunn’s test, α = 0.05).

The experiments conducted during this study have proved that the resistance of grape clusters to *P. viticola* changes considerably during their phenological development. To assess possible changes in leaf resistance during the seasonal development, leaf disc assays were performed and disease severity was determined from sporulation. These experiments showed a high *P. viticola* resistance of leaves from the cultivars Cabernet Cortis, Solaris and Souvignier Gris (Fig. 4). In contrast, the leaves of the cultivar Pinot Noir were clearly more susceptible. Leaves of Pinot Noir showed a high disease severity of 77% and 73% respectively, at the early development stages, BBCH 53–55 and BBCH 57–59. As leaves matured, resistance increased significantly. Thus, at stage BBCH 65–68, disease severity dropped to 50% and further to 16% at stage BBCH 71–73. At the beginning of ripening (BBCH 81–83), disease severity was only 3%.

Younger leaves of Cabernet Cortis and Solaris were also more susceptible than older leaves. However, compared with Pinot Noir, disease severity was significantly lower for Cabernet Cortis (13% and 6%) and for Solaris (6% and less than 1%) at BBCH 53–55 and BCCH 57–59, respectively. At later phenological stages, almost no sporulation was detectable for Cabernet Cortis and Solaris, resulting in disease severities that generally stayed below 1%. In contrast, Souvignier Gris did not show increased susceptibility on younger leaves. The infestation rate at BBCH 53–55 and BBCH 57–59 was about 2%. In later stages, the levels varied between 1–2% and thus remained at a constantly low level. Statistically significant cultivar specific differences between the susceptibility at different developmental stages were found in some cases but these differences were clearly smaller in the Pinot Noir comparison (Fig. 4B). Also, the leaves of the Pinot Noir cultivar were significantly more susceptible at each developmental stage than those of the other cultivars.

## Discussion

The aim of the present study was to develop an approach to assess the resistance characteristics of grapevine plants on both cluster and leaves during different phenological stages under standardized and reproducible conditions. Results from four consecutive years (2020–2023) proved that Pinot Noir clusters are highly susceptible until BBCH 77–79 (berry touch) and that resistance of grape clusters increases significantly with the beginning of berry ripening (BBCH 81–83). As expected, all PIWIs were clearly less susceptible than the traditional cultivar. However, this did not equally apply to all developmental stages. For example, Souvignier Gris showed the same cluster susceptibility as Pinot Noir until BBCH 75 (berries are pea-sized, bunches start to hang). The cultivars Cabernet Cortis and Solaris showed different levels of resistance during their complete phenological development. Both cultivars showed their highest susceptibility during the development of inflorescences until bloom. With the beginning of fruit development (BBCH 71–73), disease severity decreased rapidly to 20% and less. As soon as the berries were pea-sized, both Cabernet Cortis and Solaris were almost completely resistant to infections by *P. viticola*. These results indicate that the approach presented is suitable for determining the resistance of grape clusters at different stages of development.

The results may lead to the assumption that all tested PIWIs are highly susceptible to *P. viticola* until fruit development. Therefore, it is important to emphasize that the procedure presented sought to expose grape inflorescences/clusters to maximum disease pressure as afforded by ideal conditions for infection (100% relative humidity and temperatures of more than 17.5 °C)^24^. Also, applying an excess of sporangia solution directly to clusters also promoted a high number of infections^25^. Such heavy infections should be rare in the field and only occur on rainy, warm summer days with high infestation pressure inside the vineyard. In 2021, near-optimal infection conditions for downy mildew occurred in open field trial plots in Freiburg im Breisgau (Germany). Nevertheless, even these natural conditions did not lead to the high infestation rates on PIWI clusters observed on potted vines in the current study. While disease severity exceeded 90% for Pinot Noir in the above-mentioned field trial, infestation on grape clusters was significantly lower for Souvignier Gris (19%), Cabernet Cortis (6%) and Solaris (2%) (Supplemental Data 1). The trial was repeated in 2022 and 2023. However, due to the hot and dry weather conditions, no downy mildew infestation was detected on the clusters of the grape cultivars in 2022, and only a minor infestation was detected in 2023 in the open field study. The results of the field trial with untreated vines showed that all PIWIs tested in the current study were resistant under natural conditions in the vineyard. The difference between the field and potted vine trials could also be explained by physical protection inside the canopy. The significantly more resistant leaves in the fruit zone may protect the still susceptible clusters from infection. Furthermore, since most PIWIs show a high level of resistance on leaves, these can indirectly protect the clusters by keeping the inoculum in the vineyard low. Regardless, PIWI leaves tested in this study showed a high level of resistance throughout the season. This protection potential was bypassed by the direct inoculation of the clusters in the potted vines trial.

The reason for a higher susceptibility to the pathogen during early developmental stages of clusters compared to later stages is not clear. A crucial role for the onset of resistance in seasonal progression could be the transformation of functional stomata into non-functional, closed stomata, called lenticels. It was demonstrated that stomata of grape berries turn into lenticels 1–6 weeks post bloom depending on the climate and, consequently, the phenological development^26^. Hence, the traditional cultivars Riesling and Chardonnay remained susceptible to *P. viticola* until 6 weeks after bloom^26^. These observations are consistent with the result of the trials from the present study regarding cluster susceptibility of the traditional cultivar Pinot Noir. In conclusion, a decreasing number of functional stomata on grape clusters might also mediate the high level of resistance in the late phenological stages of PIWIs. Microscopic studies has shown that stomata from receptacles, pedicels of berries and rachis are collapsed or blocked in Solaris already at BBCH 69^27^. However, the early, intermediate levels of resistance found in Cabernet Cortis and Solaris in the trials with potted vines of the current study are not caused by ontogenetic resistance but may be mediated by induced resistance. Stilbene accumulation at the infection site was reported for inflorescences (BBCH 53) of Solaris, while levels were significantly lower at the later phenological stages BBCH 69 and 75^27^. Most studies on induced resistance have been conducted on leaf disc tests. For example, various defense responses have been described in PIWIs such as stilbene accumulation^28^ or necrosis, probably as a result of a hypersensitive response (HR)^29^. R-proteins of the nucleotide-binding leucine-rich repeat (NB-LRR) class, which allow plants to recognize a certain pathogen in the early stages of infection and thus trigger a cell death response, might be responsible for HR. Even though the QTLs of *Rpv1*, *Rpv3*, *Rpv10*, and *Rpv12* are associated with genes encoding R-proteins, the defense mechanisms mediated by the different *Rpv*-loci are not yet fully understood^21,30,31^. *Rpv3.1* mediated resistance is associated with the accumulation of stilbenes and programmed cell-death^32^. *Rpv10* and *Rpv12* also meditate a strong HR but seem to be faster than *Rpv3.1* plants thus preventing the development of the pathogen at an earlier stage^20^. However, the results of the present study show that the level of resistance between leaves and clusters differs considerably. Therefore, it is not clear to what extent the listed responses are responsible for the resistance of clusters. Microscopic studies of cell death response or gene expression experiments during the corresponding developmental stages of grape clusters might help to further the understanding of these observations.

PIWIs carry a great potential to reduce fungicide applications^33^. However, the underlying resistance mechanisms can be overcome under natural conditions by certain isolates of *P. viticola*^15,20^. Therefore, selective pressure on *P. viticola* should be maintained throughout the season to sustain resistance over time^17^. Besides pyramiding of *Rpv*-loci and prophylaxis by viticultural practices, fungicide treatments should be applied for this purpose. The goal of this study was to identify the most susceptible grape organs during the season in order to tailor fungicidal applications. In summary, the present method can be used to assess differences in the resistance level of grape cultivars during their phenological development. In the future, the approach could also be adapted to other grape pathogens such as powdery mildew (*Erysiphe necator*) or black rot (*Guignardia bidwellii*) in order to provide growers with precise recommendations for timing of fungicide treatments against these pathogens.

### Supplementary Information

Supplementary Information.

## Data Availability

The datasets generated during and/or analyzed during the current study are available from the corresponding author on reasonable request.
