# Supplementary material for: A method for phenotypic evaluation of grapevine resistance in relation to phenological development
Source: Sci Rep. 2024 Jan 9;14:915. doi: 10.1038/s41598-023-50666-4 (PMC10776754; doi:10.1038/s41598-023-50666-4)
Supplement: Supplementary file 1 — Supplementary Information. [file 41598_2023_50666_MOESM1_ESM.docx]

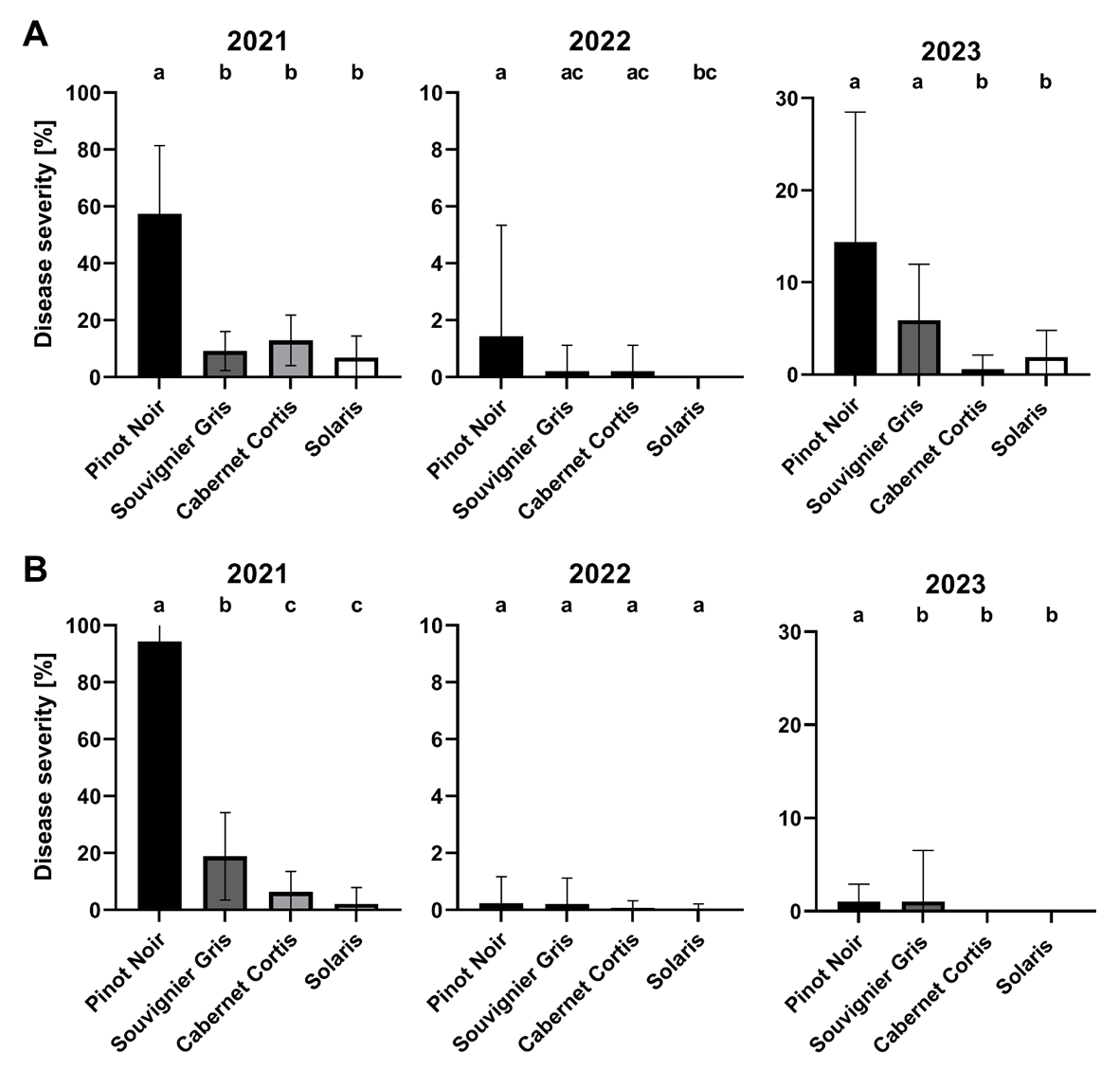
**Supplemental Data 1: Disease severity of leaves and clusters of different grapevine cultivars naturally infected with *Plasmopara viticola* in the field**

A: Assessment of downy mildew disease severity on leaves of Pinot Noir (N = 30), Souvignier Gris (N = 30), Cabernet Cortis (N = 30), and Solaris (N = 30) in 2021, 2022, and 2023.

B: Assessment of downy mildew disease severity on clusters of Pinot Noir (N = 30), Souvignier Gris (N = 30), Cabernet Cortis (N = 30), and Solaris (N = 30) in 2021, 2022, and 2023.

Graphs show the mean value of disease severity. Error bars show standard deviation, different letters indicate significant differences between the cultivars (Kruskal-Wallis test followed by multiple comparison with Dunn's test, α = 0.05).

Material & Methods:

All cultivars were planted in the same vineyard in Freiburg im Breisgau, Germany. No plant protection measures were performed in this vineyard. Evaluation of the disease was carried out at softening of berries (BBCH 85) by trained personnel of the State Institute of Viticulture and Oenology (WBI; Freiburg im Breisgau, Germany). Disease severity was rated optically with a scale from 0 % to 100 % in accordance with the standards of the European and Mediterranean Plant Protection Organization (EPPO). For this purpose, the percentage of infestation was rated for 30 leaves and 30 clusters per cultivar by visual assessment.
